# The application and influence of “Small Private Online Course” based on flipped classroom teaching model in the course of fundamental operations in surgery in China

**DOI:** 10.1038/s41598-023-50580-9

**Published:** 2024-01-03

**Authors:** Xiaoyi Zhou, Guanyu Yu, Xu Li, Wei Zhang, Xinwen Nian, Jin Cui, Xianzhao Wei, Yu Sun

**Affiliations:** 1 Teaching and Research Section of Surgery and Field Surgery, First Affiliated Hospital of Navy Medical University, Shanghai, 200433 China; 2 Department of Orthopaedics, First Affiliated Hospital of Navy Medical University, Shanghai, 200433 China; 3 Department of Colorectal Surgery, First Affiliated Hospital of Navy Medical University, Shanghai, 200433 China; 4 Department of Urology Surgery, First Affiliated Hospital of Navy Medical University, Shanghai, 200433 China; 5 Department of Burn Surgery, First Affiliated Hospital of Navy Medical University, Shanghai, 200433 China

**Keywords:** Health occupations, Medical research

## Abstract

To investigate the effect of “Small Private Online Course” (SPOC) based on flipped classroom teaching model on the students in the course of fundamental operations in surgery. A prospective study. 8-year program students (juniors) majored in clinical medicine in Navy medical university. The mastery of theoretical knowledge and operational skill of the students, the comparison of final test examination score between traditional teaching method and “SPOC + flipped classroom” model and the feedback completed by students. Our study found that SPOC + flipped classroom could significantly increase the efficacy of the class and enhance the ability of the students compared with the traditional method. The new teaching model could have a positive influence for medical students on their basic knowledge and operational skill.

## Introduction

With the rapid development of network technology, an increasing number of countries began to try informatization and integration of educational resources^1^. Hence, the occurrence of massive open online courses (MOOCs) in 2012 has reformed traditional teaching boundaries in the past few years^2^. However, after years of exploration and development, there were still several major problems for MOOCs: 1. High registration rate but also high dropout rate; 2. Absence of interaction and communication between teachers and students; 3. High cost of maintenance for the MOOC platform^3^.

In order to address the disadvantages of MOOC, Pro. Fox proposed the idea of Small Private Online Course (SPOC) in 2013^4^. Supported by advanced teaching conception and network technique, the application of SPOC acted as a teaching method for supplement rather than substitution of class teaching. Instead of full-time online teaching in MOOC, SPOC combined online resources and class teaching, allowing face-to-face communication between students and teachers as well as students and students^5^.

Fundamental operations in surgery is a bridge course connecting theoretical knowledge with practical operation^6^. Traditionally, teachers would take a 40-min academic class and then begin to practical operation on animals in the following class. One of the major problem of this teaching model was that students could not completely understand the whole procedure of the required surgery and figure out the operational details only through one 40-min theory class, which would affect the surgery on animal later^7^.

The combination of SPOC and flipped classrooms provided a new vision on promoted course teaching and elevated efficiency in class^8^. Students were required to self-study operational videos recorded by teachers before each class. The academic class was replaced by question-and-answer session. The saved time was given back to students so that they could have more time for practice operation on animals. It has been found that “SPOC + flipped classroom” teaching model could help students absorb relevant theoretical knowledge and promote learning attitudes and efficiency^9^. However, the effect of SPOC + flipped classroom on the course of fundamental operations in surgery is still unknown in China.

Therefore, the present study was conducted to apply SPOC based on flipped classroom model in the course of fundamental operations in surgery and investigate the effect of this new teaching method on the students.

## Results

### The evaluation of class performance (theoretical knowledge and operational skill)

**Figure 1:**
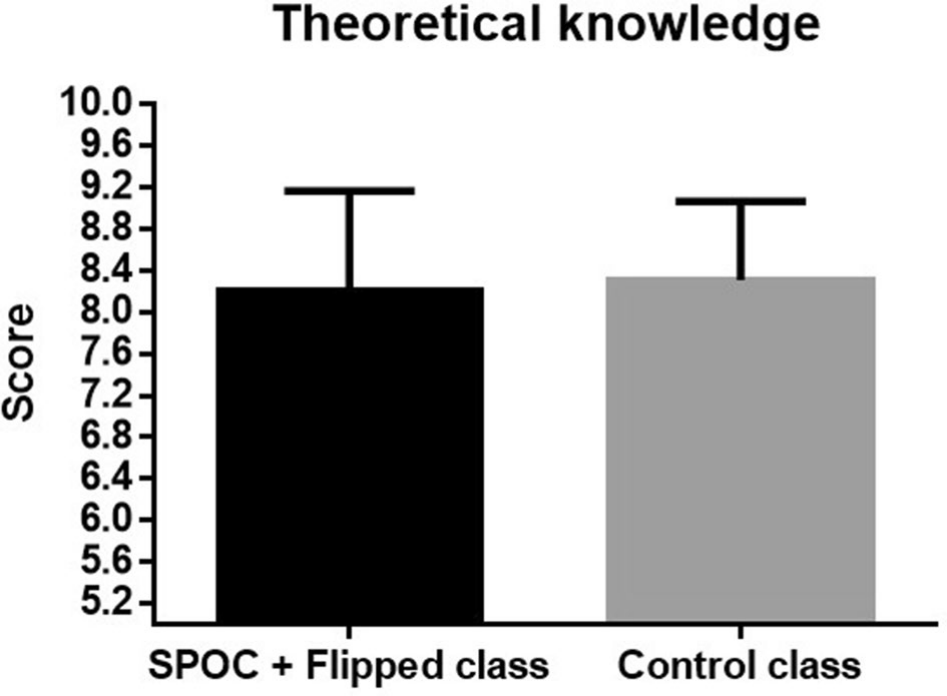
Comparison of the score of theoretical knowledge between two groups rated by teachers.

**Figure 2:**
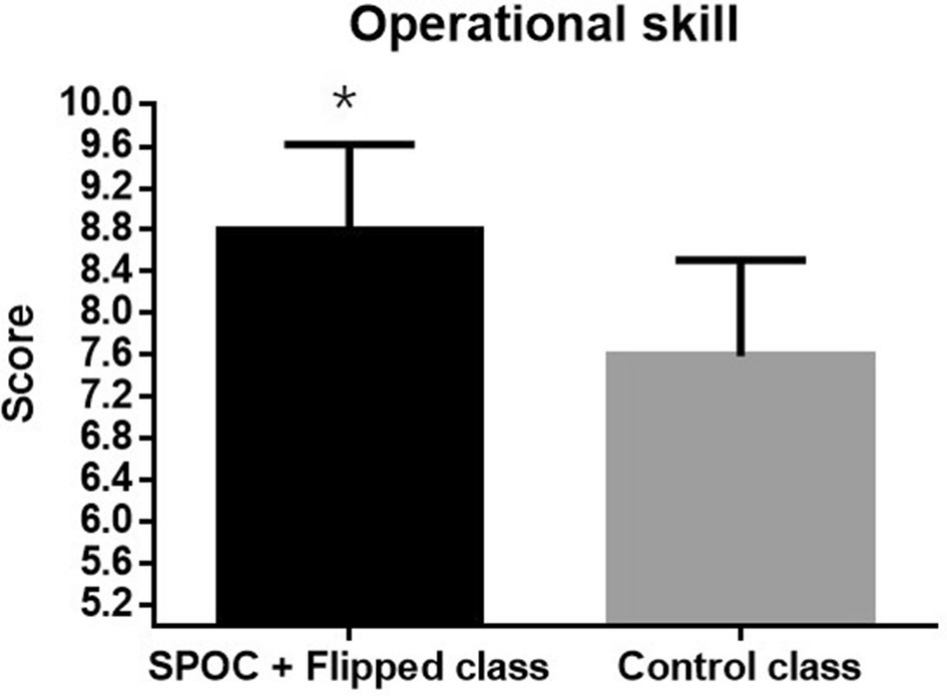
Comparison of the score of operational skill between two groups rated by teachers.

Theoretical knowledge of each student was evaluated by teacher during operation in practice class (Table 1 and Figs. 1, 2). Results showed that there was no significant difference between two different group for the mastery of basic knowledge (8.20 ± 0.96 vs 8.31 ± 0.75, P = 0.49). However, score of operational skill of the students in the SPOC + flipped class was significantly higher than control group (8.79 ± 0.81 vs 7.58 ± 0.91, P < 0.01), indicating that SPOC + flipped class teaching model could improve the capacity of the students during the operation.

**Table 1:** Class performance of the students in SPOC + flipped class and control class evaluated by teachers.

	SPOC + flipped class	Control class	*t*	*P*
Theoretical knowledge	8.20 ± 0.96	8.31 ± 0.75	− 0.68	0.49
Operational skill	8.79 ± 0.81	7.58 ± 0.91	7.69	< 0.01

### The distribution of final score

**Figure 3:**
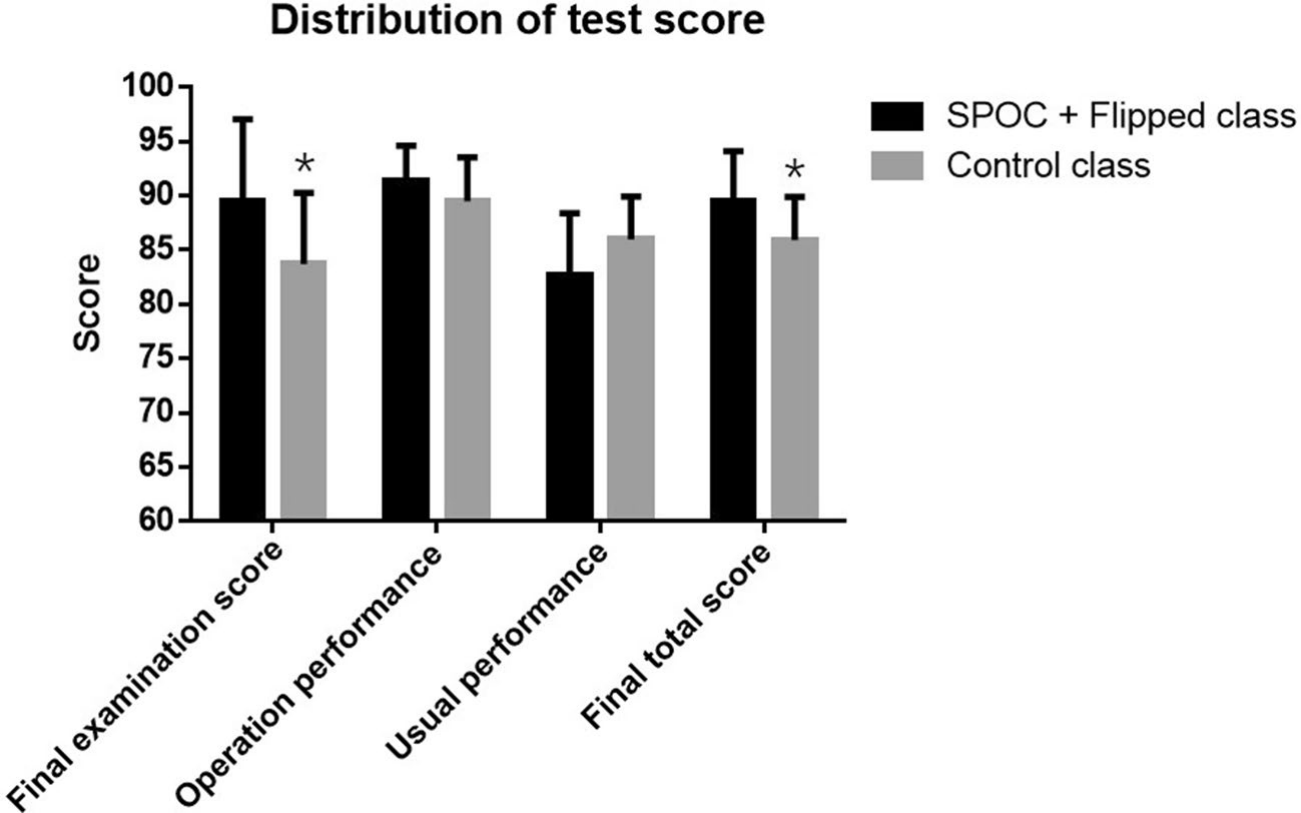
Comparison of the distribution of final test score between “SPOC + flipped class” group and control class.

As was demonstrated in Table 2 & Fig. 3, the final examination score of the students in “SPOC + flipped class” group was 89.50 ± 7.56, while the score in control class was 83.72 ± 6.56. The operation performance evaluated by teachers was 91.37 ± 3.25 in experimental class vs 89.51 ± 4.04 in control class. The score of usual performance in experimental class was 82.76 ± 5.65 vs 85.97 ± 3.97 in control class. After converting into the final score, The final total score was 89.57 ± 4.56 in the “SPOC + flipped class” and 85.93 ± 3.97 in the traditional teaching class. The results revealed that “SPOC + flipped class” significantly improved the final examination score. Similarly, “SPOC + flipped class” increased the score of operation performance and final total score, but with no significant difference.

**Table 2:** The distribution of final score in two different classes.

	SPOC + flipped class	Control class	T	*P*
Final examination score	89.50 ± 7.56	83.72 ± 6.56	4.48	< 0.01
Operation performance	91.37 ± 3.25	89.51 ± 4.04	2.79	0.06
Usual performance	82.76 ± 5.65	85.97 ± 3.97	0.14	0.88
Final total score	89.57 ± 4.56	85.93 ± 3.97	4.67	< 0.01

### Students’ feedback on “SPOC + flipped classroom”

The results of questionnaire were demonstrated in Table 3. Most of the student (96.9%) were satisfied with the experience of this course and they admitted that “SPOC + flipped classroom” could enhance their ability of basic knowledge and operative skills (92.2%). Besides, the preclass video helped them a lot to understand the key points of the operation accurately (93.7%). Many students felt passionate in the course (85.9%) and would like to recommend this course to others (89.1%). This teaching model was suitable for students (92.2%).

**Table 3:** Questionnaire on the students’ feedback on the course.

Item	Question	Yes	No
Learning satisfaction and students’ feeling on “SPOC + flipped classroom”			
1	I am interested and pleased with the experience of this course	62 (96.9%)	2 (3.1%)
2	I feel enthusiastic and passionate in this course	55 (85.9%)	9 (14.1%)
3	I think the “SPOC + flipped classroom” teaching model is quite suitable for me	59 (92.2%)	5 (7.8%)
The influence of “SPOC + flipped classroom” on students			
4	“SPOC + flipped classroom” enhances my ability of basic knowledge and operative skills	59 (92.2%)	5 (7.8%)
5	The preclass videos helped me understand the key points and knowledge of the operation accurately and clearly	60 (93.7%)	4 (6.3%)
6	I would like to recommend others to take this course	57 (89.1%)	7 (10.9%)

## Discussion

As the development of university education, a more flexible and refined course model like SPOC is believed to be “an inevitable evolution” by many educational experts^10^. The first appearance of SPOC in China could be traced back to 2019. And for now, there has been no research investigating the effect of SPOC + flipped classroom in the course of fundamental operations in surgery in China. The teaching implementation using SPOC model relied on high quality video resources^11^. Thus, in the present study all the teachers prepared a high-definition video specific to each surgery for students to learn before each class. These videos contain preparation, detailed operation and cautions of each surgery, so that the students could not only gain theoretical knowledge but also study practical procedures.

Here “Small” means that the number were strictly limited from tens to hundreds of students in the course. And “Private” means that the students in the class should meet the access requirement, most of whom are students at school. The students in SPOC are more homogeneous than those in MOOC, such as their age, region, self-learning capacity et al. These characteristics of SPOC result in more interaction compared with MOOCs. And it would enhance the engagement and efficacy of the course^12^.

In the present study, we found that SPOC + flipped class teaching model could significantly improve the operational capacity during the operation compared with the control class evaluated by our teachers. SPOC provided high quality teaching resource and students were asked to carefully preview the contents before the class. We found that the flipped classroom method based on SPOC could significantly improve learning outcomes compared with traditional learning method. The total final score increased from 87.7 to 90.2 compared with two different teaching methods. Similarly, Zhang et al.^13^ found that application of SPOC into flipped classroom increased students’ scores in the preclass test (65.13 ± 12.45 vs 53.46 ± 8.09) and postclass test (80.43 ± 14.29 vs 69.01 ± 12.81) in the physiology course. SPOC + flipped class enabled teaching process no longer being constrained by traditional method, but giving students sufficient time to absorb knowledge and thinking. The video lecture before the class allowed the students self-learning without the restriction of limited class time and class environments. The process of self-study before class prolonged the study duration and thus promoted the whole study efficiency. Likewise, Hu et al.^14^ investigated the influence and effect on “SPOC + flipped classroom” on physical education students’ learning motivation and found that it had a positive impact of the video lectures on student’s motivation on basketball skills and promoted their motivation autonomy. The conception of SPOC claimed for independent study. This student-centered teaching model activated their learning potential and helped them to combined theory with practice in the class time.

In order to arouse students’ autonomy, we planned to skip the theoretical class and directly entered into practical class in the course of fundamental operations in surgery. A 40-min class was saved for extent learning and operation. This is one of the advantages of SPOC + flipped classroom, which converts the knowledge imparting part from classroom to online course, and give students more time to ask questions and communicate with teachers and perform operation in the following class. The teachers acted as the role of directors and every 4 students formed a surgical group to complete the operation cooperatively. The students had to independently understand the principle and operational procedure during the whole operation. Thus, careful preview was quite necessary before the class in order to solve the possible problems they might met during the operation. Sufficient preclass preparation gave the students positive feedback and made them feel competent and passionate about the surgery. This might be one reason that the students in “SPOC + flipped class” could get a higher score in operative skill rated by teachers in the present study.

According to self-determination theory, three basic demands must be satisfied so as to get high quality development of self-realization and motivation: demand for autonomy, demand for competence and demand for relatedness^15^. In the present study, the students carried out operations based on the knowledge which took place before class, making them feel that the procedure and operation was easier and more understandable. The students enhanced their sense of ability during the process of preparation before class and the operation in the class. In accordance with our finding, Guo et al.^16^ provided a blended learning model via SPOC in embryology and found that integrated SPOC with face to face lectures was a more effective means for teaching compared with the traditional lecture-based teaching model. Knowledge reserve in the students in SPOC + flipped class would significantly promote the interaction between students and teacher in the class. And teachers could spend more time specifically on explaining and discussing the difficulties and key points of the course, which was one of the most obvious advantages compared with traditional teaching method.

There were several limitations in the present study. First, the COVID-19 pandemic disrupted and delayed the implementation of teaching intervention. Second, the number of the students enrolled in the present study were restricted for the teaching method of SPOC. Last but not least, with the development of advanced teaching tools, substitutions like anthropomorphic dummy, stimulated properties and models and virtual reality could be applied in this course. In some class, these modern tools could replace living animals like beagle, which is our future direction.

## Conclusion

“SPOC + flipped classroom” teaching model could have a positive influence for medical students on their basic knowledge and operational skill. Moreover, the results showed that most students were interested and satisfied with this teaching model. These results indicated that the application of “SPOC + flipped classroom” could enhance the engagement and enthusiasm of the students and promote the effect on the course of fundamental operations in surgery.

## Materials and methods

### Participants

This was a prospective study and the students enrolled in the present study were all 8-year program juniors majored in clinical medicine. The course of fundamental operation was carried out in two continuous academic years (2021&2022). The experimental group (SPOC + flipped classroom) in the present study were 8-year program students (n = 64) in the 2022 academic year in navy medical university. The control group (n = 58) enrolled 58 8-year program students (traditional teaching method) in the 2021 academic year. The entrance test score between the two grades was similar and with no significant difference. The control group was taught with lectures before operation without watching videos. The informed consent was obtained from all subjects and/or their legal guardian(s). This research has been performed in accordance with the Declaration of Helsinki and all methods were performed in accordance with the relevant guidelines and regulations. This course and study was approved by the Institutional Ethics Review Board of the Naval Medical University.

### “SPOC + flipped classroom” model design

In the pre-class stage, teachers optimized learning contents and recorded teaching video for each class. All the teaching videos were uploaded to a self-made APP by teachers. Students were asked to study the video themselves before the class and finish a quiz at the end of the video.

In the class stage, students started operation on beagle dogs directly without theoretical lecture. Teachers acted as reminders and inspectors and provided proper guidance during their operation.

**Figure 4:**
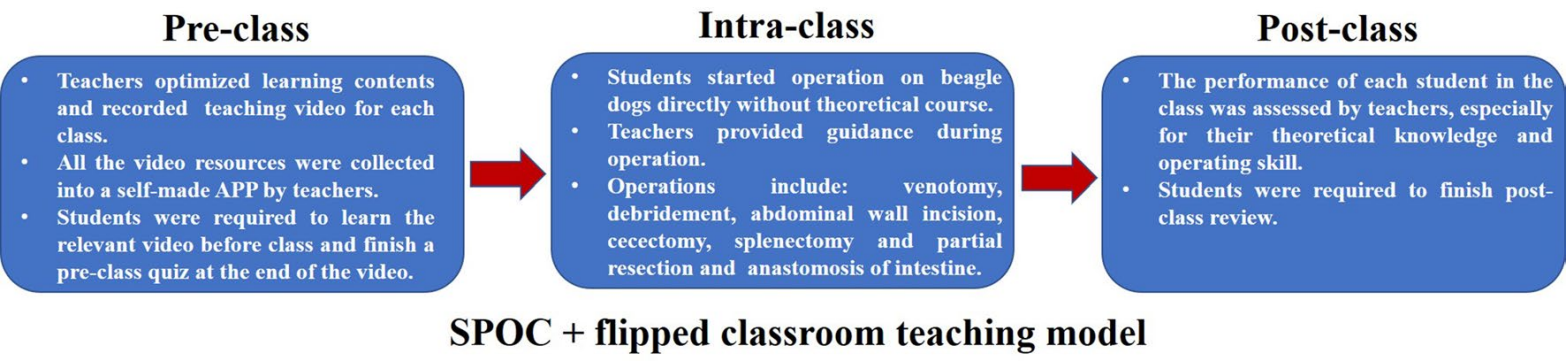
“SPOC + flipped classroom” model design.

In the post-class stage, students were required to finish post-class review. And the performance of each student during operation was rated by teachers based on theoretical knowledge and operating skill. (Fig. 4).

### Evaluation of theoretical knowledge and operational skill along with the class as usual performance by teachers

During each class, teachers would evaluate the mastery of theoretical knowledge and operational skill of each student by rating each item from 0 to 2. As for theoretical knowledge (Table 4), teachers would prepare several questions such as the anatomical features (important vessel, organ, nerve, et al.) surrounding the surgical site, indications for appendectomy or debridement and so on. As for the operational skill (Table 5), we divided each section into several simple actions such as incision, exposure, ligation, suture, knot, use of vessel forceps, scissors, needle holders, et al. The total score of 0 represented that the student did not know anything before the class, while the total score of 10 represented well command of knowledge and skill for the current surgery. All the teachers were trained before this course. The inter-rater reliability between different teacher was acceptable. The reliability and validity of two scales were evaluated and confirmed before the present study.

**Table 4:** Basic theoretical knowledge scale.

Content	0	1	2
1.Understanding of topographic anatomy surrounding the surgical site			
2.Understanding the indication and contraindication of the surgery			
3.Understanding the procedures of the surgery			
4.Understanding the key points which should be paid attention on during the surgery			
5.Complications might happen postsurgery and the treatment principles			
Total point

**Table 5:** Operational skill scale.

Content	0	1	2
1. Standard and correct surgical operation			
2. Overall operation proficiency			
3. Proficiency of using surgical instruments			
4. Awareness of sterility and humanity during surgery			
5. Ability of teamwork			
Total point			

### Comparison of final test examination score between traditional teaching method and “SPOC + flipped classroom” model

After finishing all the class, the students were taking the final theoretical and practical examination. The final examination paper was consisted of 5 major parts (choice question*20, gap filling*10, noun explanation*5, short answer question*5 and operation record*1). Intestinal anastomosis was performed as the final operation performance test. The score of operation performance was rated by teachers under unified standards. The final total score was composed of examination paper score (50%), operation performance (40%) and usual performance (10%, consist of students’ classroom performance during each class, such as attendance rate, interaction activeness and surgery record after each operation).

### Feedback of “SPOC + flipped classroom” by students

A 6-item questionnaire was designed to collect the feedback of this new teaching method in the “SPOC + flipped classroom” group (Table 3). The questionnaire was delivered and completed after the last class. The questionnaire could be divided into two major parts: 1. Learning satisfaction and students’ feeling on “SPOC + flipped classroom”; 2. The influence of “SPOC + flipped classroom” on students.

### Data analysis

SPSS22.0 was used to conduct data analysis. All the data was expressed as means ± SD. A *t* test was performed to compare mean score of class performance and final test examination between two different teaching models. P < 0.05 was consider as statistically significant difference.

### Supplementary Information

Supplementary Information 1.

Supplementary Information 2.

## Data Availability

All data generated or analysed during this study are included in this published article [and its supplementary information files].
